# Synthesis and Characterization of Dental Nanocomposite Resins Reinforced with Dual Organomodified Silica/Clay Nanofiller Systems

**DOI:** 10.3390/jfb14080405

**Published:** 2023-08-01

**Authors:** Maria Saridou, Alexandros K. Nikolaidis, Elisabeth A. Koulaouzidou, Dimitris S. Achilias

**Affiliations:** 1 Laboratory of Polymer and Color Chemistry and Technology, Department of Chemistry, Aristotle University Thessaloniki, 541 24 Thessaloniki, Greeceaxilias@chem.auth.gr (D.S.A.); 2 Division of Dental Tissues’ Pathology and Therapeutics (Basic Dental Sciences, Endodontology and Operative Dentistry), School of Dentistry, Aristotle University Thessaloniki, 541 24 Thessaloniki, Greece; koulaouz@dent.auth.gr

**Keywords:** dental nanocomposite resins, quaternary ammonium compounds, organomodified nanosilica, clay nanoparticles, nanotechnology

## Abstract

Quaternary ammonium (QA) compounds have been widely studied as potential disinfectants in dental restorative materials. The present work investigates whether the gradual displacement of nanosilica by QA-clay nanoparticles may have an impact on the physicochemical and mechanical properties of dental nanocomposite resins. For this purpose, Bis-GMA/TEGDMA-based composite resins were initially synthesized by incorporating 3-(trimethoxysilyl)propyl methacrylate (γ-MPS)-modified nanosilica/QA-clay nanoparticles at 60/0, 55/5, 50/10, 40/20, and 30/30 wt% filler loadings. Their structural characterization was performed by means of scanning electron microscopy (SEM) and X-ray diffraction analysis (XRD). The degree of double bond conversion (DC) over time and the polymerization shrinkage were determined with Fourier transform infrared spectroscopy (FTIR) and a linear variable displacement transducer (LVDT), respectively. Mechanical properties as well as water sorption and solubility parameters were also evaluated after storage of nanocomposites in water for 7 days at 37 °C. Spectral data revealed intercalated clay configurations along with areas characterized by silica-clay clusters for clay loadings up to 30 wt%. Furthermore, the insertion of 10 wt% QA-clay enhanced the auto-acceleration effect also sustaining the ultimate (DC), reduced the setting contraction and solubility, and, finally, yielded flexural modulus and strength very close to those of the control nanocomposite resin. The acquired results could herald the advanced design of dental restorative materials appropriate for contemporary clinical applications.

## 1. Introduction

Over the last decades, dental composite resins have been widely applied in both anterior and posterior tooth restorations. Aesthetic characteristics and toxicity reasons associated with amalgam fillings have strongly motivated the dental industry to promote composite resins to direct restorations [1]. Nevertheless, the oral cavity constitutes a dynamic regime governed by high masticatory stresses in combination with several biological and chemical threats that can affect the longevity of dental composites [2,3]. To overcome these limiting factors, composites’ formulations are usually manipulated by the utilization of dimethacrylate monomers such as Bis-GMA, UDMA, and TEGDMA reinforced with diverse inorganic fillers that are conjugated with the polymer matrix through silane coupling agents. Nowadays, contemporary trends involve dental composite resins reinforced with zirconia [4,5], silica nanoparticles [6], halloysite nanotubes [7], and even composites derived from biomass rice husk silica that demonstrate excellent performance [8]. Although fillers with specific size distribution can contribute to the achievement of good mechanical and physicochemical performance, dental composites are still a challenge with secondary caries originating from the metabolic activity of pathogenic bacteria [9].

Several approaches have been adopted in order to enhance the microbial resistance of dental restorative materials. Although the direct incorporation of leachable disinfectants [10] such as fluoride [11], chlorhexidine (CHX) [12], benzalkonium chloride [13], essential oils [14], and a wide range of nanoparticles [15] into dental composites can induce antimicrobial ability in the composite, there are still some ambiguities related to loss activity, weakening of the remaining restoration, and discoloration over time [16]. Furthermore, mesoporous silica nanoparticles loaded with CHX might reinforce the dental composite against plaque formation, but these may also affect its mechanical performance due to the release and recharge mechanism of the biocide agent [17]. Considering the exploitation of biocide agents through such nanoscale vehicles, their unstable position is attributed to the physical absorption of nanoparticles within the polymer network. Other well-established approaches involve the incorporation of non-leachable quaternary ammonium monomers (QA) [18,19], or QA combined with a nanofiller such as silica (QASi) [20,21], clay (QA-clay) [22], or in the form of polymeric QA polyethyleneimine particles (QPEI) [23]. In this manner, it is believed that functional groups of QA are chemically bound to the organic matrix, and the antimicrobial component remains entrapped in the polymer network of the dental composite, thus ensuring an optimum antibacterial action over time [24].

To the best of our knowledge, there is no literature data describing dental composite resins reinforced with both potentially antimicrobial QA-clay nanofillers [25,26] and silica nanoparticles sustaining the overall physicomechanical performance [27,28]. The aim of this study was to investigate the effect of the displacement of nanosilica by QA-clay nanoparticles on the physicochemical and mechanical properties of the obtained dental nanocomposite resins. For this purpose, experimental dental nanocomposites based on a BisGMA/TEGDMA matrix and filled with 3-(trimethoxysilyl)propyl methacrylate (γ-MPS) modified nanosilica were initially synthesized as control samples (golden standards). Silica nanoparticles were then replaced by different types of QA-clays at different filler loadings to produce pure nano-filled resin composites. The null hypothesis was that the type and amount of nanoclay might influence the structural configurations and final properties of the developed nanocomposites. The obtained results are supposed to be conducive to the design of contemporary dental nanocomposite resins, meeting the requirements of modern clinical applications.

## 2. Materials and Methods

### 2.1. Materials

**Scheme 1:**
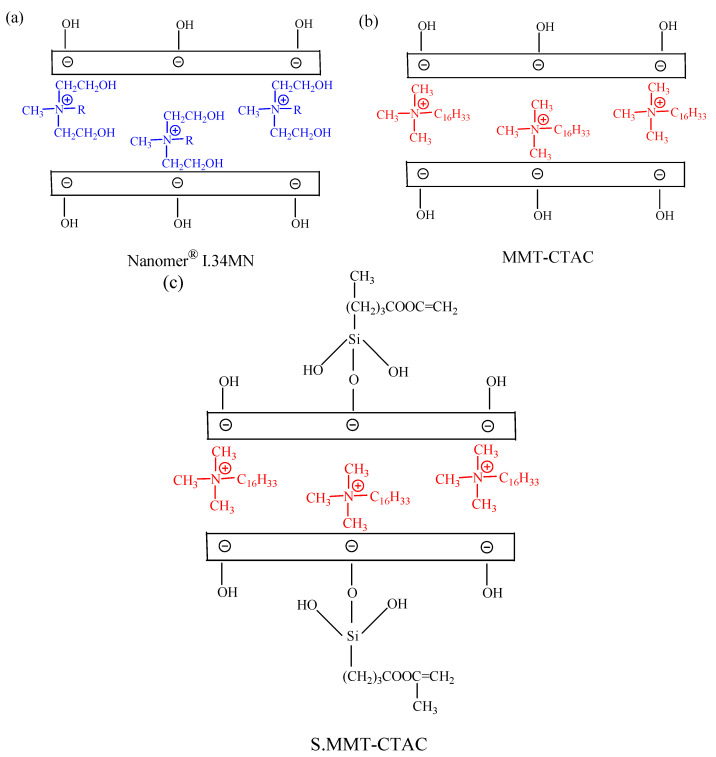
Structural configurations of: (**a**) Nanomer^®^ I.34MN; (**b**) MMT-CTAC; (**c**) S.MMT-CTAC clay used for the synthesis of dental nanocomposite resins (R stands for hydrogenated tallow).

The monomers triethylene glycol dimethacrylate (TEGDMA), 95%, and 2,2-Bis[p-(2′-hydroxy-3′-methacryloxypropoxy)phenylene]propane (Bis-GMA) were both provided by SIGMA-ALDRICH CHEMIE GmbH (Steinheim, Germany). The co-initiator 2-(dimethylamino)ethylmethacrylate (DMAEMA), 99%, and initiator camphorquinone, 98%, were purchased from J&K Scientific GmbH (Pforzheim, Germany). Organically modified silica nanopowder (S.MPS) with 3-(trimethoxysilyl)propyl methacrylate (γ-MPS) was synthesized and subsequently characterized in our previous work [29]. The commercially available organomodified montmorillonite Nanomer^®^ I.34MN, i.e., an –onium ion modified clay containing 25 to 30 wt% methyl dixydroxyethyl hydrogenated tallow ammonium ion (QA-clay), was produced by Nanocor Company (Hoffman Estates, IL, USA) and supplied by Aldrich (Taufkirchen, Germany). Two experimental QA-clays, namely, a clay type intercalated with cetyltrimethylammonium chloride (MMT-CTAC), as well as its surface-modified analog with 3-(trimethoxysilyl)propyl methacrylate (S.MMT-CTAC), were both prepared and characterized in our previous works [22,30]. The particular structures of the above QA-clays are presented in Scheme 1. All other chemicals used were of reagent grade.

### 2.2. Preparation of the Uncured Dental Composite Pastes

Seven groups of experimental composites were prepared by initially mixing a Bis-GMA/TEGDMA base (50:50 wt/wt) that contained camphorquinone (0.2 wt%) and DMAEMA (0.8 wt%) as a photo-initiating system. Afterward, neat S.MPS and nanoparticle combinations of S.MPS/Nanomer^®^ I.34MN, S.MPS/MMT-CTAC, and S.MPS/S.MMT-CTAC were individually inserted in the resin by manual mixing until the powder was completely wetted with organic matrix, and the obtained mixture was ultrasonicated for 10 min. Environmental conditions were kept constant (22 °C, 40% relative humidity, 1 atm) during the mixing process. The specific composition of each prepared nanocomposite is described in Table 1. The overall nanofiller loading was kept at 60 wt% to ensure paste handling properties almost similar to a commercial dental composite resin.

### 2.3. Measurements

#### 2.3.1. Structural Characterization of the Experimental Dental Resins

A JEOL JSM-6390LV (JEOL USA, Inc., Peabody, MA, USA) scanning electron microscope system (SEM) operated within 0.5–30 kV (3 nm resolution) was utilized in order to examine the surface of the prepared materials. A protective carbon black coating against charging was applied on the sample surfaces prior to the scanning process. Microphotos were taken by focusing electron beams on the studied surfaces and using Smile Shot^TM^ software. Elemental analysis of the samples was performed by means of the INCAPentaFETx3 (Oxford Instruments, Abingdon, UK) energy-dispersive X-ray (EDX) microanalytical system, and the percentage of the targeted elements was determined by averaging different areas acquisitions.

A Miniflex II X-ray diffractometer (XRD) supplied by RigakuCo. (Tokyo, Japan) was used to record the spectral patterns of the obtained nanocomposites within the scanning range (2 theta) of 2–12°. The following operation conditions were selected: Cu Ka radiation with wavelength 0.154 nm, angle steps of 0.05°, 5 s/angle step.

#### 2.3.2. Setting Contraction Kinetics

The “bonded-disk” technique established by Watts et al. [31,32,33] was applied in order to calculate the real-time contraction due to polymerization process. Particularly, an unpolymerized sample in the form of a disk (1.0 × 8.0 mm) was placed on the surface of a glass plate (3 mm thickness) within a mounted metal ring (internal diameter ~15 mm). A flexible membrane was used to adhere the upper surface of the sample. A uniaxial transducer (LVDT) was placed on the center of the membrane, in order to measure the linear variable displacement of the specimen over time. A dual system combining a transducer indicator (E 309, RDP Electronics Ltd., Wolverhampton, UK) and a high-resolution analog-to-digital converter (ADAM-4016) was utilized to transmit the signal from the LVDT module to the computer. The data were acquired by means of AdvantechAdam/Apax.NET Utility software (version 2.05.11). An LED light-curing device (Bluephase^®^ Style M8, Ivoclar Vivadent AG, FL-9494, Schaan, Liechtenstein) with an intensity of 800 mW·cm^−2^ ± 10% was positioned above the glass plate to continuously irradiate the unset samples for 5 min. A radiometer (Hilux, Benlioglu Dental Inc., Ankara, Turkey) was utilized to check the output intensity of the photo-curing unit. Environmental conditions were kept constant (22 °C, 40% relative humidity, 1 atm) during the photo-curing process. Each dental nanocomposite was measured five times (n = 5). The calculation of the strain (%) was based on the following formula:

(1) $$\varepsilon \left(\%\right)=100\times \frac{\Delta L}{L_{0}}$$

where ε (%) represents the strain (%), and DL (mm) and L_0_ (mm) are the deformation due to setting and the initial thickness of the nanocomposite, respectively.

#### 2.3.3. Degree of Conversion

Polymerization kinetics were performed by placing a small amount of each composite between two translucent Mylar strips, which were pressed to produce a very thin film. The upper surface of the films of unpolymerized composites was exposed to visible light of the above LED unit and directly measured by a FTIR spectrometer (Spectrum One, PerkinElmer Inc., Waltham, MA, USA) at diverse setting moments (0, 5, 10, 15, 20, 25, 30, 40, 60, 80, 120, 180 s). Spectral acquisition of the nanocomposite films (n = 5) was conducted in the wavenumber region of 4000–600 cm^−1^, performing 32 scans per sample at 4 cm^−1^ resolution. Environmental conditions were kept constant (22 °C, 40% relative humidity, 1 atm) during the photo-curing process. The obtained records were manipulated with specific software (Spectrum v5.0.1, Perkin-Elmer LLC 1500F2429). The degree of conversion (DC%) over time was calculated based on the area of the absorption band at 1637 cm^−1^ (aliphatic C=C bonds) and the band at 1580 cm^−1^ (aromatic C=C bonds) as well as on the best fit baseline method according to the Beer–Lambert law [34]. For this purpose, the following equation was used:

(2) $$DC\left(\%\right)=\left[1-\frac{{\left(\frac{A_{1637}}{A_{1580}}\right)}_{polymer}}{{\left(\frac{A_{1637}}{A_{1580}}\right)}_{monomer}}\right]\times 100$$

#### 2.3.4. Mechanical Properties

A Teflon mold (2 × 2 × 25 mm) was filled with the unset material, in order to prepare bar-shaped specimens for three-point bending tests according to the ISO 4049. Each side of the mold was irradiated for 40 s with the aforementioned LED lamp, by overlapping the unset areas. Environmental conditions were kept constant (22 °C, 40% relative humidity, 1 atm) during the photo-curing process. Ten specimens (n = 10) per dental resin were formed and subsequently immersed in deionized water for 1 week at 37 ± 1 °C. Then, they were placed on a three-point bending module between two supports of 20 mm distance. The bend tests were conducted in a universal testing machine (Testometric AX, M350-10 kN, Testometric Co., Ltd., Rochdale, UK at a cross-head speed of 0.5 mm·min^−1^). The exerted load (N) versus deformation were recorded, and the acquired data were manipulated with the WinTestAnalysis CX software (Version 3.5.30.10). The flexural modulus (E, GPa) and strength (σ, GPa) were calculated based on the following formulae:

(3) $$E=\frac{F_{1}l^{3}}{4bd_{1}h^{3}}10^{-3}\;and\;\sigma =\frac{3F_{max}l}{2bh^{2}}$$

where F_1_ is the measured load (N), F_max_ is the highest load measured prior to mechanical failure (N), l is the distance between the supports (20 mm), b is the width of the sample (mm), h is the height of the sample (mm), and d_1_ is the deformation (mm) related to the load F_1_.

#### 2.3.5. Water Sorption and Solubility

The determination of the water sorption and solubility parameters was performed in accordance with the requirements of ISO 4049. A Teflon mold was filled with each unset dental nanocomposite, in order to produce disc-shaped specimens (15 mm × 1 mm). The specimens (n = 5) were set with the aforementioned LED curing unit by irradiating for 40 s each side and targeting to nine preselected overlapping irradiation areas. Environmental conditions were kept constant (22 °C, 40% relative humidity, 1 atm) during the photo-curing process. The discs were stored in a desiccator, and then they were pre-conditioned at 37 ± 1 °C for 24 h. Afterwards, the specimens were transferred into another desiccator (23 ± 1 °C) to remain for 2 h. Then, they were weighed (±0.1 mg accuracy) by means of an analytical balance (Sartorius TE 124S, Sartorius AG, Goetingen, Germany). The above procedure was repeated until the materials reach a constant mass (*m*_1_). Their volume (*V*) was calculated upon their mean thickness and diameter, which were previously measured with a digital micrometer (±0.02 mm accuracy). Subsequently, the specimens were stored in water at 37 ± 1 °C. After 1 week, they were isolated, left to dry and weighed (*m*_2_). The discs were then placed in desiccator until a constant mass (*m*_3_) was achieved.

The water sorption parameter, *W_sp_* (μg/mm^3^), was calculated from the formula:

(4) $$W_{sp}=\frac{m_{2}-m_{3}}{V}$$

where *m*_2_ is the mass of the disc (μg) after storage in water for 1 week, *m*_3_ is the mass of the reconditioned disc (μg), and *V* is the volume of the disc (mm^3^).

The solubility parameter, *W_sl_* (μg/mm^3^), was calculated according to the following equation:

(5) $$W_{sl}=\frac{m_{1}-m_{3}}{V}$$

where *m*_1_ is the conditioned mass (μg) of the disc before the storage in water.

#### 2.3.6. Statistical Analysis

The assumption of normal distribution was investigated for the variables using the Shapiro–Wilk test. One-way analysis of variance (ANOVA) was used to compare the flexural modulus, sorption, solubility, and strain between the seven groups. The Kruskal–Wallis test was used to compare flexural strength between the seven groups. Bonferroni corrections were made to adjust for multiple tests. Statistical analysis was performed using IBM SPSS Statistics 28. The statistical significance level was set at *p*-value ≤ 0.05.

## 3. Results and Discussion

### 3.1. Limitations of Research

In the framework of the evaluation of the developed dental nanocomposite resins, certain possible restrictions affecting the research outputs could be considered. The common hand spatulation mixing method was applied to prepare the starting nanocomposite paste. The above technique may have an effect on the extent of fillers’ dispersion and on the overall performance of the obtained composite. Moreover, the prevention of bias could not be supported by randomization methods, and the determined sample size for the majority of the conducted tests was mainly dictated by ISO 4049 “Dentistry-Polymer Based Restorative Materials”. The above limitations mainly originate from the limited availability of the experimentally synthesized silica and clay nanoparticles used in composites’ formulations. As the current research targeted the basic requirements of ISO 4049, which are always necessary for the dental industry, antimicrobial and cytotoxicity tests were not conducted in parallel. Future experimental cycles would provide particular data in order to estimate the potential antimicrobial effect of the proposed dental nanocomposite resins due to the presence of the QA-clays utilized here.

### 3.2. Morphological Characterization of the Prepared Materials

**Figure 1:**
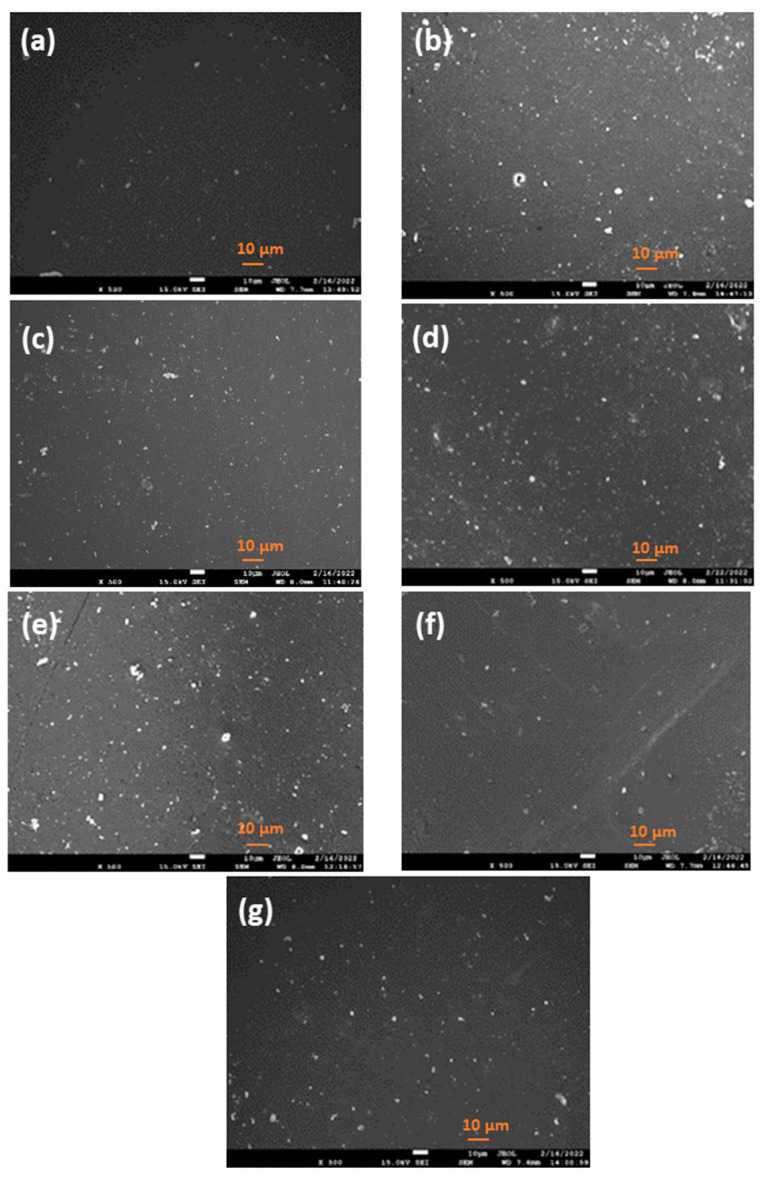
SEM microphotos taken for dental nanocomposites reinforced with: (**a**) pure nanosilica (S.MPS); (**b**–**e**) Nanomer^®^ I.34MN clay particles (5–30 wt%) in the presence of nanosilica; (**f**) MMT-CTAC clay; (**g**) S.MMT-CTAC clay nanoparticles (10 wt%) in the presence of nanosilica.

SEM images captured for the produced dental nanocomposite resins are presented in Figure 1. Regarding the composite loaded with 60 wt% S.MPS nanoparticles (Figure 1a), it is apparent that an adequate dispersion of silica was accomplished (white spots) into the formed polymer network (dark regions), even if some small aggregates can be discriminated. Similar clustering tendencies have also been observed by other researchers and ascribed to the widely applied hand spatulation mixing technique of monomers with inorganic fillers [35,36,37,38,39]. The desirable distribution of the total filler content seems to be retained when the amount of Nanomer^®^ I.34MN clay was elevated up to 30 wt% (Figure 1e). A stepwise size increment of the created agglomerates is also observed in the range of 10–30 wt% clay (Figure 1c–e). The aforementioned behavior could be attributed to the intermolecular hydrogen bonding between the intercalating agents of the hydrophylic Nanomer^®^ I.34MN particles. On the other hand, more bulky agglomerates occurred for the S.MPS/Nanomer 55/5 (Figure 1b), denoting not only the equivalent clay–clay interactions but also the possible formation of silica-clay clusters due to the hydrogen bonding interactions between the free unreacted hydroxy groups originating from the organomodified silica and the hydroxy groups of the Nanomer^®^ I.34MN quaternary ammonium intercalant (Scheme 1). Furthermore, the insertion of 10 wt% MMT-CTAC (Figure 1f) and S.MMT-CTAC clay (Figure 1g) in the dimethacrylated organic matrix can also lead to sufficient dispersion of the used nanoparticles similar to that of S.MPS/Nanomer 50/10. In particular, the filler agglomeration detected for the S.MPS/S.CTAC 50/10 nanocomposite maybe due to the side reactions of the double bonds attached on the S.MMT-CTAC and/or the double bond reactions between both the silane modified silica and clay nanofillers (Scheme 1).

Table 2 encompasses the data derived from the EDX spectra of the studied dental nanocomposites. A constant enrichment of nanocomposites with Al is observed by increasing the content of Nanomer clay from 5 to 30 wt%, thus ensuring the good dispersion of clay particles into the formed polymer network over the tested filler concentration range. The Al content also remained for composites with 10 wt% MMT-CTAC and S.MMT-CTAC at higher levels than Nanomer, probably due to the occurrence of larger agglomerates revealed by SEM images. Under this regime, the accumulation of Al species could be favored, resulting in their high abundance over the scanned surfaces.

**Figure 2:**
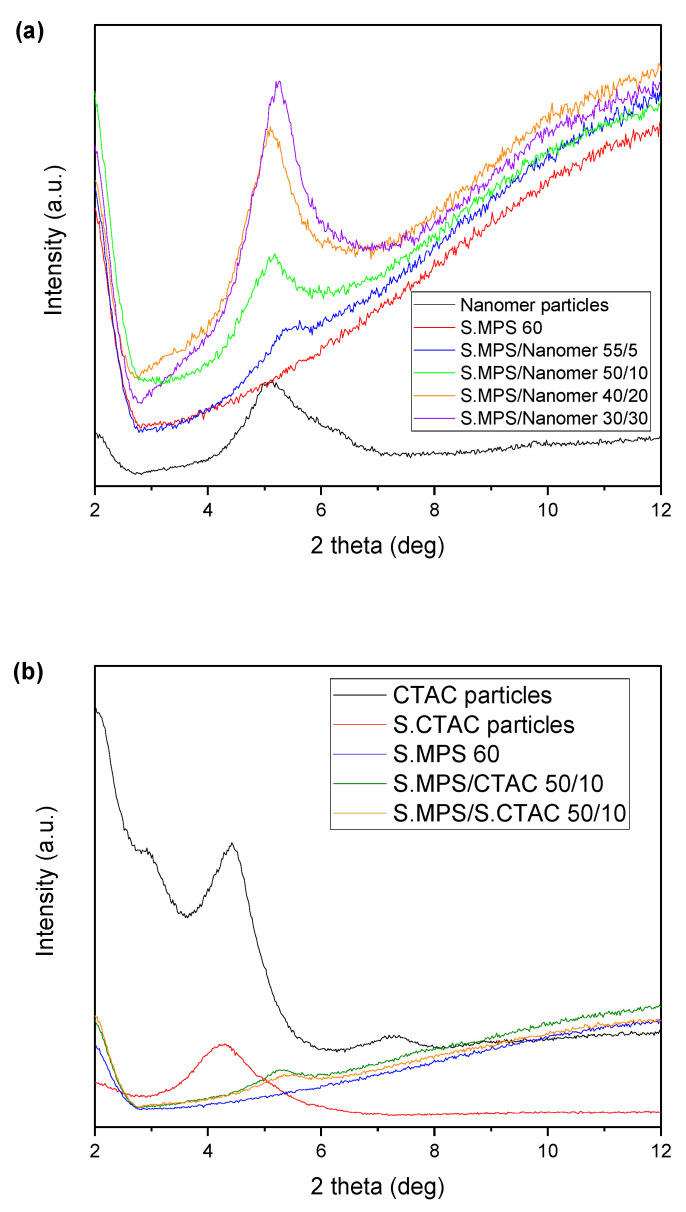
Comparative XRD spectra of the investigated dental nanocomposite resins filled with nanosilica and: (**a**) Nanomer ^®^ I.34MN clay (5–30 wt%); (**b**) MMT-CTAC and S.MMT-CTAC clay nanoparticles at 10 wt% filler loading.

The XRD spectra recorded for the synthesized dental nanocomposite resins are illustrated in Figure 2. It is observed that even though clay nanoparticles exhibit diffraction peaks at 2*θ* = 5.14° (Nanomer^®^ I.34MN clay, Figure 2a), 4.42°, and 4.31° (MMT-CTAC and S.MMT-CTAC, Figure 2b), their corresponding S.MPS nanocomposites always display X-ray diffractions at higher angles. Provided that amorphous nanosilica develops diffuse scattering at the angular region of 20–25° [40], possible cluster formation through amorphous silica–Nanomer^®^ I.34MN interactions could be responsible for the shifts in clay reflections (001) to higher 2*θ* values. These interactions seem to be even stronger when the clay loading remains at 5–10 wt%, as the dominant silica nanoparticles (55–50 wt%) clearly impede the diffraction pattern of clay, regardless of the chemical structure of the intercalating agent (Figure 2a,b). Further incorporation of clay nanoparticles up to 30 wt% gradually enhances the peak intensities of dental composites, thus implying the amplified presence of intercalated clay configurations (Figure 2a). Although the intercalation of macromolecular chains within clay galleries governs the morphological characteristics of the synthesized dental nanocomposites, there might still be some areas characterized by silica-clay clusters over the nanocomposite structures. These findings could be also supported by the SEM observations, as previously described.

### 3.3. Polymerization Reaction Kinetics

**Figure 3:**
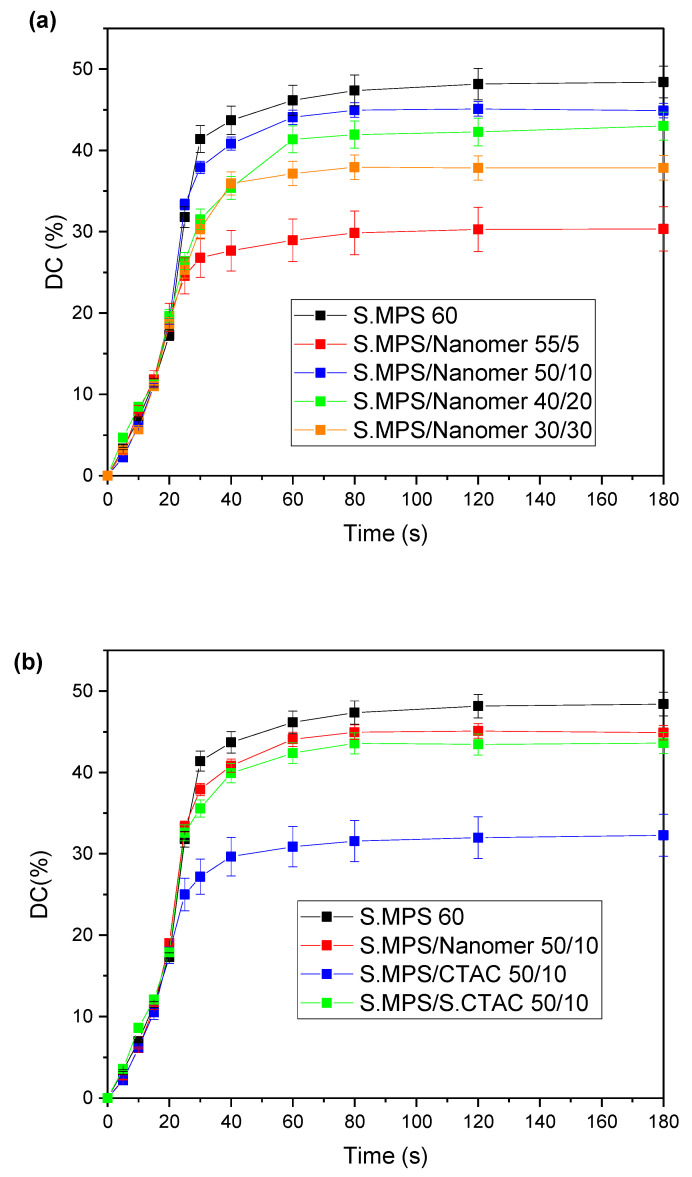
Plots of degree of conversion (DC%) versus time for dental nanocomposites containing: (**a**) different silica/clay nanoparticle ratios (up to 30/30 wt%); (**b**) diverse types of nanoclay at silica/clay nanoparticle ratio 50/10 wt%.

Figure 3 illustrates the degree of conversion versus time curves recorded for dental nanocomposites filled with silica-clay nanoparticles. The DC (%) values calculated after 180 min curing are listed in Table 3. Concerning the different clay mass fraction, the plots of the free radical polymerization kinetics revealed the occurrence of the auto-acceleration or gel effect phenomenon as it was mainly expressed by a steep increase in the slope mostly in the range of 20–25 min of the setting procedure (Figure 3a). In general, the gel effect is controlled by diffusion phenomena [41], where the free volume inside the developed polymer network can be limited due to the presence of inorganic fillers along with the developed macromolecular chains. Under such circumstances, the mobility of macroradicals is restricted, leading to further difficulty in their finding each other and terminating the polymerization reaction. Hence, their local concentration is increased, resulting in the augmentation of the reaction rate. The auto-acceleration phenomenon was found to be stronger for the S.MPS 60 and S.MPS/Nanomer 50/10 nanocomposites, approaching DC values of (17.31–31.8%) and (19.01–33.54%), respectively. This attitude was attenuated almost sequentially for S.MPS/Nanomer 40/20 (19.66–26.18%), S.MPS/Nanomer 30/30 (18.67–25.30%), and S.MPS/Nanomer 55/5 (19.46–24.39%) composites. The most comprehensive distribution of nanoclay into the organic matrix that was proved by SEM for S.MPS/Nanomer 50/10 nanocomposite could better constrain the macroradical movement due to the decrease in the total free volume of the polymer network, and it could extensively sustain the auto-acceleration phenomenon. The DC value found for S.MPS 60 nanocomposite (48.41%, Table 3) was comparable to 46.84% reported by Liu et al. for Bis-GMA/TEGDMA-based composites containing 30 wt% silica nanoparticles [42]. Wang et al. also described dental resin composites filled with 60 wt% silica nanoclusters exhibiting 49.8% [43]. Moreover, it is obvious that the step-by-step enrichment of dental composites with 10 to 30 wt% Nanomer clay leads to the descending of double bond conversion from 44.89 to 37.86% (Table 3). The relatively low determined degree of conversion could be attributed to the glass effect phenomenon occurring when the extensive network has been developed, thus provoking the high viscosity of the reaction mixture. The phenomenon is controlled by the limited diffusion of both the monomers and the initial initiator radicals and finally affects the rate constant of chain propagation [41,44,45,46]. In addition, the DC value for S.MPS/Nanomer 55/5 was further lowered to 30.34%. Regarding the S.MPS/Nanomer 40/20, S.MPS/Nanomer 30/30, and S.MPS/Nanomer 55/5 composites, the SEM findings denoted larger silica-clay aggregates that might act as microfillers, affecting the light absorption and scattering, and thus weakening the light photo-initiation process [47]. The gel effect phenomenon was also observed for S.MPS/S.CTAC 50/10 and S.MPS/CTAC 50/10 nanocomposites during the period of time from 20–25 min (Figure 3b). Particularly, the S.MPS/S.CTAC 50/10 yielded almost similar conversions (17.89–32.59%) as the S.MPS/Nanomer 50/10 composite during auto-acceleration phenomenon, whereas lower DC values (17.96–24.96%) were calculated for S.MPS/CTAC 50/10. The vinyl functionalities attached on the surface of S.MMT-CTAC nanoparticles (Scheme 1) could account for the comparatively enhanced DC throughout the gel effect, as methacrylate groups of clay might react with those originated from Bis-GMA and TEGDMA monomers. The ultimate DC measured for S.MPS/S.CTAC 50/10 (43.61%) was close to that of S.MPS/Nanomer 50/10, and for S.MPS/CTAC, the DC was found to be 32.28% (Table 3). The absence of any functional groups in the structure of MMT-CTAC clay (Scheme 1) in combination with some nanofiller agglomeration (Figure 1f) may be responsible for the weaker glass effect phenomenon during the photo-polymerization of the S.MPS/CTAC composite.

### 3.4. Water Sorption and Solubility

Water sorption of the composite resin materials constitutes a physicochemical process of clinical importance due to its significant impact on strength and wear performance [48]. The weight values per volume unit of the obtained nanocomposites after aging in water for 7 days are listed in Table 3. Statistically significant differences (*p* < 0.05) between the seven studied groups are also included. Regarding the increasing nanoclay content in dental nanocomposite resins, it is shown that the sorption characteristics are influenced by the gradual addition of Nanomer ^®^ I.34MN clay, reaching experimental values in the range of 28.48–44.97 μg/mm^3^. The composite reinforcement with clay nanofiller up to 20 wt% yields *W_sl_* results that conform with the sorption requirements of ISO 4049 (<40 μg/mm^3^) [49]. Janda et al. highlighted the correlation between the filler loading of resin-based filling materials and water sorption [50]. It seems that the hydrophilic nature of Nanomer^®^ I.34MN clay promotes the attraction of water molecules via hydrogen bonding as the clay amount becomes larger. Consequently, the penetration of water proceeds intensively and, hence, affects the sorption resistance of the highly reinforced nanocomposites. Moreover, the S.MPS/S.CTAC 50/10 nanocomposite exhibited a remarkable resistance against water sorption (31.59 μg/mm^3^) in comparison to the S.MPS/Nanomer (33.37 μg/mm^3^) and S.MPS/CTAC (41.12 μg/mm^3^) counterparts. In that case, the surface silanized S.MMT-CTAC clay could serve as a supplementary crosslinking agent that further enhances the polymer network against the entry of water molecules.

The solubility of dental composite resins is closely related to the release of unreacted monomers from the polymer matrix to the aqueous environment during the aging process conducted at 37 ^ο^C, which somehow simulates the oral conditions. All the solubility data presented in Table 3 are in accordance with the solubility criteria of ISO 4049 (<7.5 μg/mm^3^) [49]. Statistically significant differences (*p* < 0.05) are also included in Table 3. Indeed, the incorporation of 5 wt% Nanomer ^®^ I.34MN clay does not vitally affect the ability of the material to withstand the elution of the entrapped monomers from the crosslinked polymer structure to the outer aqueous environment. It is worth pointing out that the S.MPS/Nanomer 50/10 presented better resistance (4.79 μg/mm^3^) when compared to the control sample S.MPS 60 (6.21 μg/mm^3^). Al-Shekhli et al. reported solubility results for the commercial dental composite resins Premise, Herculite, and Supreme XT (4.36, 4.02 and 4.02 μg/mm^3^) that are relatively close to that of S.MPS/Nanomer 50/10 [51]. The relatively high DC value found for the S.MPS/Nanomer 50/10 (44.89%) and the well-dispersed intercalated clay platelets (Figure 1c and Figure 2a) capable of acting as barriers against the diffusion of the remaining monomers may account for the observed low solubility level. Although the additional clay loading can render the nanocomposite more susceptible to monomer release, the determined solubility values did not exceed the corresponding values of the control dental nanocomposite. S.MPS/CTAC 50/10 and S.MPS/S.CTAC 50/10 composites also mitigated the monomer migration to the aqueous medium, as revealed by their measured solubilities (5.72 and 3.02 μg/mm^3^). In particular, both the DC found for the S.MPS/S.CTAC dental nanocomposite resin (43.61%) and the sufficient dispersion of the silicate layers into the polymer network (Figure 1g) may account for the performance mentioned above.

### 3.5. Polymerization Shrinkaze Kinetics

**Figure 4:**
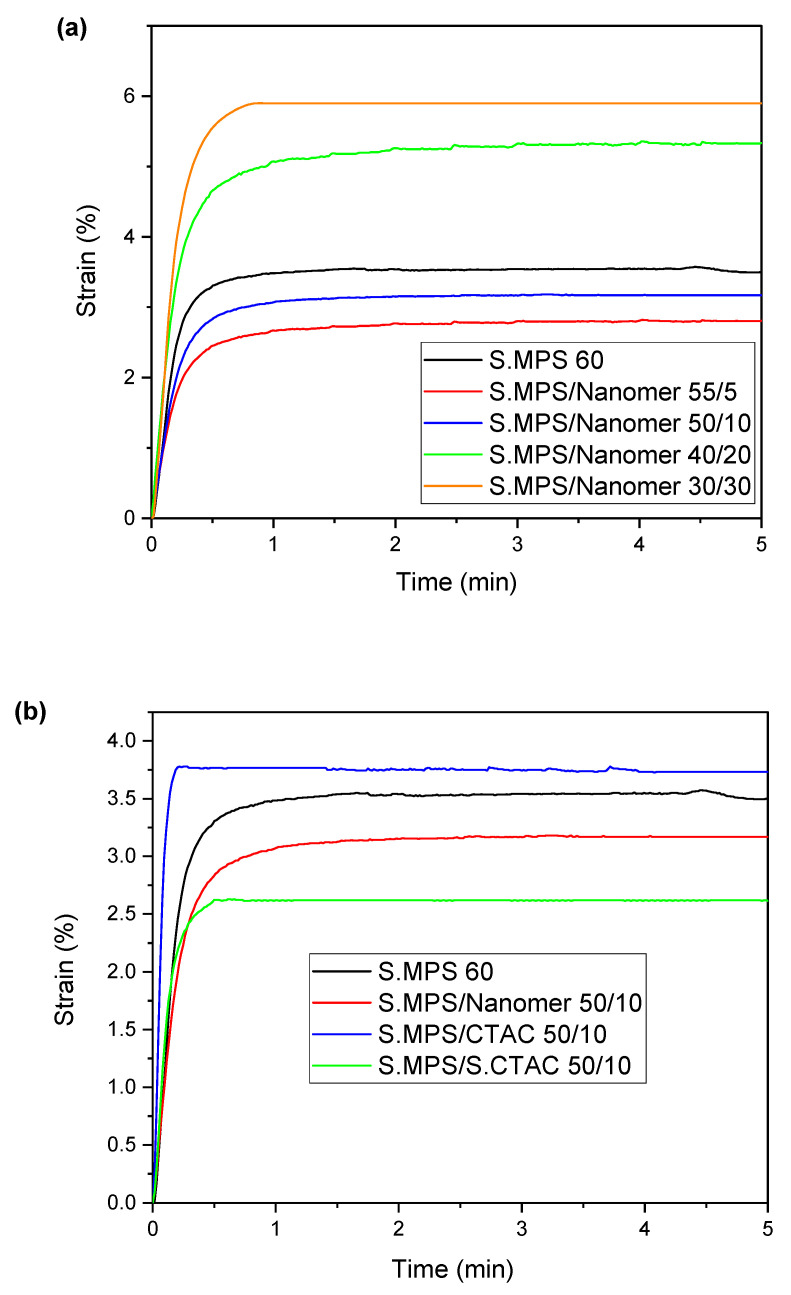
Time dependence of polymerization shrinkage of the studied dental nanocomposites reinforced with: (**a**) specific silica/clay nanoparticle ratios (up to 30/30 wt%); (**b**) several types of nanoclays at silica/clay nanoparticle ratio 50/10 wt%.

Figure 4 depicts the kinetics of polymerization shrinkage for the total of the synthesized dental nanocomposite resins. In addition, Table 3 summarizes the strain values (%) calculated for the studied materials, also involving statistically significant differences (*p* < 0.05) between the seven studied groups. It is well accepted that light-cured composite resins are challenged with dimensional alterations caused by polymerization reactions of the organic monomers [52]. The process involves the conversion of van der Waals interactions into covalent bonds, resulting in a decrease in free volume, internal stresses, and, finally, in the formation of microleakage at the tooth restoration interface [33]. The variations in the filler type may have a strong effect on the polymerization shrinkage–stress kinetics of resin composites [53]. According to Figure 4, an abrupt increase in the strain curves at the early stages of the setting procedure (<10 min curing) was recorded for all studied materials, and this trend was more intense for S.MPS/Nanomer 30/30 (Figure 4a) and S.MPS/CTAC 50/10 (Figure 4b). Regarding the Nanomer^®^ I.34MN clay incorporation, the lowest setting contraction was determined for the S.MPS/Nanomer 55/5 composite (2.76%), followed by S.MPS/Nanomer 50/10 (2.93%), which are limited in comparison to S.MPS 60 (3.51%). Organomodified clay nanoparticles are known to swell through the insertion of the developed macromolecular chains between silicate lamellae, leading to the increment in the internal free volume of the clay and eventually to the decrease in the total strain of the nanocomposite [54,55]. However, the higher mass fractions of clay at 20 and 30 wt% resulted in the ultimate strains of 5.22% and 6.14%, respectively. The possible formation of inorganic agglomerates into the intercalated nanocomposite structures, as previously supported by SEM and XRD results, might decrease the expansion and free volume of Nanomer^®^ I.34MN clay and subsequently the polymerization shrinkage of the cured nanocomposite. Regarding the different type of clay at 10 wt% content, the S.MPS/CTAC nanocomposite experienced larger setting contraction (3.68%) than S.MPS/Nanomer, possibly due to the different degree of clay swelling effect. On the contrary, the S.MPS/S.CTAC 50/10 composite displayed the highest dimensional stability (2.63%), perhaps owing to the higher extension of clay platelet disorientation, as proved by the lowest-intensity peak in the XRD diffractogram (Figure 2b). This configuration could contribute to the increase in the free volume of S.MMT-CTAC clay and maintain the nanocomposite’s polymerization shrinkage at a relatively low level.

### 3.6. Flexural Properties

**Figure 5:**
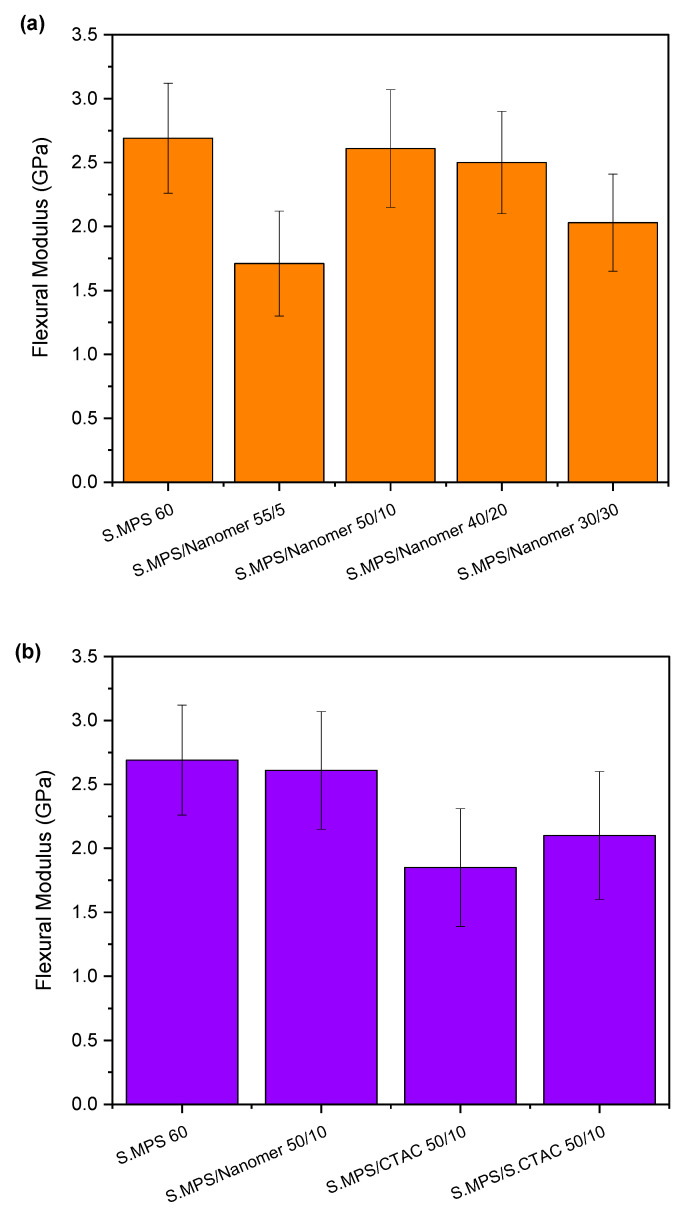
Flexural modulus of the synthesized dental nanocomposite resins nanofilled with silica and: (**a**) Nanomer^®^ I.34MN clay at different nanoclay loadings (5–30 wt%); (**b**) different organoclay types at 10 wt% concentration.

The flexural modulus tendencies determined for dental nanocomposites containing silica along with clay nanoparticles are presented in Figure 5, and the corresponding values involving statistically significant differences (*p* < 0.05) are listed in Table 4. Regarding the variation in clay loading (Figure 5a), it is clear that the displacement of the total content of silica nanoparticles by 10 wt% nanoclay in the dental composite formulation can retain the stiffness of the ultimate nanocomposite (2.61 GPa) at a similar level as the control S.MPS 60 (2.69 GPa). These findings are very close to the elastic modulus assessed by Atali et al. for the universal resin composites Omnichroma (2.87 GPa) and Admira Fusion x-tra (2.33 GPa) [56]. Further incorporation of clay nanoparticles may lead to a stepwise decrease in modulus up to 22%, corresponding to the elasticity attitude of S.MPS/Nanomer 30/30 (2.03 MPa), and the largest part of this descent (19%) is due to the transition from the S.MPS/Nanomer 40/20 (2.50 GPa) to the S.MPS/Nanomer 30/30 nanocomposite. Though there is information in the literature correlating the elastic modulus with the DC [57], the aforementioned trend could be attributed to the decrease in DC values (Table 3) by clay loading elevation from 10 to 30 wt%. In addition, the increase in sorption values implies a possible plasticizing effect of water molecules during the aging of specimens, which expands the macromolecular chains of the network, resulting in the observed flexural modulus results. Figure 5b shows that at 10 wt% amount of nanoclay, the rigidity of dental nanocomposite resin with Nanomer^®^ I.34MN particles is at least 20–29% higher than the corresponding resins with MMT-S.CTAC (2.10 GPa) and MMT-CTAC nanofillers (1.85 MPa). The highest DC value determined for S.MPS/Nanomer 50/10 (44.89%) could justify the improvement in the flexural modulus versus S.MPS/S.CTAC 50/10 and S.MPS/CTAC 50/10 composites. Despite the above differences, it was also found that the presence of MMT-S.CTAC (10 wt%) can sustain the final stiffness of the nanocomposite between the values achieved when the Nanomer^®^ I.34MN was used at 20 and 30 wt% mass fractions.

**Figure 6:**
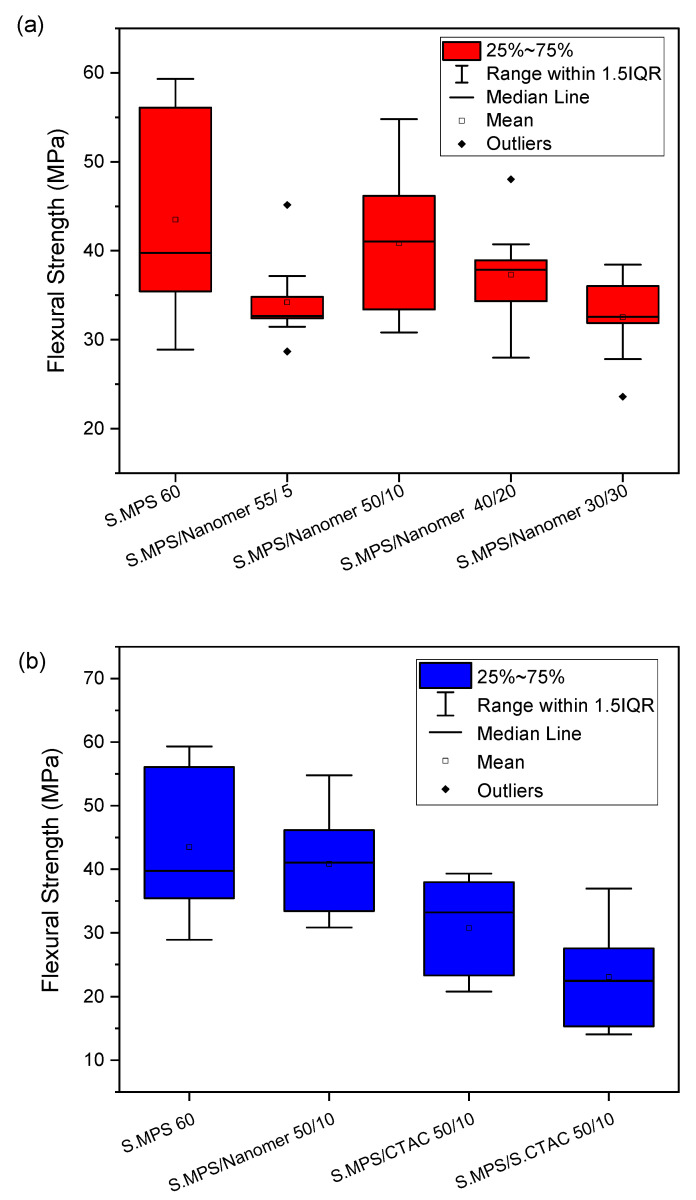
Flexural resistance of the synthesized dental nanocomposite resins nanofilled with silica and: (**a**) Nanomer clay at different nanoclay loadings (up to 30 wt%); (**b**) different organoclay types at 10 wt% concentration.

Figure 6 shows that the flexural strength is also vitally influenced by the incorporation of the different amount and type of organoclay nanoparticles. The determined values for each nanocomposite along with statistically significant differences (*p* < 0.05) are presented in Table 4. The majority of the assessed strength values were found to be superior to that of 25 MPa for dental resin composites filled with 60 wt% S.MPS nanoparticles, as mentioned by Rodriguez et al. [49]. Herein, the experimental procedure for the estimation of the nanocomposites’ mechanical behavior involved 7 days of aging in aqueous medium, so as to better simulate the oral conditions and intensify the penetration effectiveness of water molecules within the polymer network. On the contrary, other studies applied shorter aging time periods, thus presenting higher flexural strength values [28,58]. In particular, dental nanocomposites filled with 10 wt% Nanomer^®^ I.34MN clay (Figure 6a) exhibited almost the same resistance against flexural stresses (41.04 MPa) when compared with the conventional nanocomposite S.MPS 60 (39.75 MPa), followed by the slightly weaker S.MPS/Nanomer 40/20 (37.88 MPa), and, finally, the S.MPS/Nanomer 30/30 dental composite (32.57 MPa). Moreover, the descending strength of S.MPS/Nanomer 55/5 (32.66 MPa) is similar to that of S.MPS/Nanomer 30/30. The combination of SEM and XRD data could support the latter mechanical position, as the presence of clay-clay agglomerates and silica-clay clusters can result in irregular distribution of flexural stresses, thus accumulating them in limited sites, which contributes to crack propagation (Figure 1 and Figure 2). In terms of the different types of nanoclay (Figure 6b), it seems that at relatively low clay loading levels (10 wt%), the flexural strength level is better sustained for the S.MPS/CTAC 50/10 (33.32 MPa) in comparison to the composite resin containing MMT-S.CTAC (22.46 MPa). Furthermore, the S.MPS/Nanomer 50/10 dental nanocomposite resin exhibits a 23% and 82% improvement in ultimate strength in relation to S.MPS/CTAC 50/10 and S.MPS/S.CTAC 50/10 nanocomposites. The extremely weaker resistance of the latter nanocomposite resin can be explained by the filler aggregated units detected by the SEM technique (Figure 1).

## 4. Conclusions

Herein, novel dental nanocomposite resins were synthesized by incorporating organically modified silica along with QA-clay nanoparticles at different clay loadings. It was proved that both the different type and the amount of nanoclay might influence not only the structural characteristics but also the physicochemical and mechanical properties of the obtained nanocomposites, thus confirming the null hypothesis. More particularly, SEM and XRD results showed the intercalation of macromolecular chains within clay galleries, along with some areas characterized by silica-clay clusters over the nanocomposite structures, when the amount of clay reached up to 30 wt%. The degree of conversion, setting contraction, and sorption parameters of the obtained nanocomposite resins were found to decrease by elevating the nanoclay concentration. In particular, the utilization of 10 wt% Nanomer^®^ I.34MN amplified the auto-acceleration effect within the 20–25 min of setting reaction, also keeping the obtained final DC value at almost 45%. The aforementioned mass fraction of clay also decreased the shrinkage strain (2.93%) and solubility (4.79 μg/mm^3^), without affecting the flexural modulus (2.61 GPa) and ultimate strength (41.04 MPa) of the dental nanocomposite resin. These findings are expected to provide scientific information to the dental industry in terms of the correlations between the structural configurations and the ultimate properties of modern dental nanocomposite resins based on novel nanofiller systems. The awareness of the aforementioned results in combination with a comprehensive future study evaluating dental nanocomposites’ antibacterial activity could contribute to the integrated design of multifunctional dental restorative materials for modern clinical applications.

## Figures and Tables

**Table 1:** Abbreviated names of the studied materials based on their particular composition.

Dental Nanocomposite Resin	S.MPS (wt%)	Nanomer^®^ I.34MN (wt%)	MMT-CTAC (wt%)	S.MMT-CTAC (wt%)
S.MPS 60	60	-	-	-
S.MPS/Nanomer 55/5	55	5	-	-
S.MPS/Nanomer 50/10	50	10	-	-
S.MPS/Nanomer 40/20	40	20	-	-
S.MPS/Nanomer 30/30	30	30	-	-
S.MPS/CTAC 50/10	50	-	10	-
S.MPS/S.CTAC 50/10	50	-	-	10

**Table 2:** Content of clay elements (%) in dental nanocomposite resins measured by means of EDX.

Dental Nanocomposite Resin	Si (%) Mean (SD)	Al (%) Mean (SD)	O (%) Mean (SD)
S.MPS 60	46.74 (0.00)	-	53.26 (0.00)
S.MPS/Nanomer 55/5	46.33 (0.08)	0.47 (0.09)	53.20 (0.01)
S.MPS/Nanomer 50/10	46.05 (0.13)	0.78 (0.15)	53.16 (0.02)
S.MPS/Nanomer 40/20	44.55 (0.41)	2.48 (0.46)	52.97 (0.05)
S.MPS/Nanomer 30/30	43.30 (0.17)	3.90 (0.19)	52.80 (0.02)
S.MPS/CTAC 50/10	45.37 (0.28)	1.56 (0.31)	53.07 (0.04)
S.MPS/S.CTAC 50/10	45.62 (0.21)	1.27 (0.24)	53.11 (0.03)

**Table 3:** Curing kinetics data and polymerization shrinkage, solubility, and sorption values calculated for the synthesized dental nanocomposite resins. Different superscript indicates statistically significant difference (*p* < 0.05).

Dental Nanocomposite Resin	DC (%) Mean (SD)	Strain (%) Mean (Min, Max)	Sorption, Wsp (μg/mm^3^) Mean (Min, Max)	Solubility, Wsl (μg/mm^3^) Mean (Min, Max)
S.MPS 60	48.41 (1.94)	3.51 (3.31, 3.75) ^a^	27.54 (24.82, 30.37) ^a,b,g^	6.21 (4.77, 7.18) ^a,b^
S.MPS/Nanomer 55/5	30.34 (2.73)	2.76 (2.52, 2.93) ^a^	28.48 (26.84, 30.38) ^b,c^	6.61 (6.12, 7.20) ^a,b^
S.MPS/Nanomer 50/10	44.89 (0.90)	2.93 (2.43, 3.19) ^a^	33.77 (31.16, 38.47) ^c,d,g^	4.79 (4.59, 4.97) ^a,b^
S.MPS/Nanomer 40/20	43.00 (1.72)	5.22 (4.20, 6.00) ^b^	37.07 (30.38, 40.13) ^d,f^	5.67 (2.55, 9.35) ^a,b^
S.MPS/Nanomer 30/30	37.86 (1.51)	6.14 (5.09, 7.39) ^b^	44.97 (41.83, 46.13) ^e,f^	7.27 (5.48, 10.00) ^a^
S.MPS/CTAC 50/10	32.28 (2.58)	3.68 (3.40, 3.92) ^a^	41.12 (40.12, 41.81) ^f^	5.72 (4.79, 7.05) ^a,b^
S.MPS/S.CTAC 50/10	43.61 (1.31)	2.63 (2.50, 2.79) ^a^	31.59 (30.41, 33.39) ^g^	3.02 (1.62, 4.39) ^b^

**Table 4:** Mean values (SD) of flexural modulus and median values (interquartile range, IQR) of strength as measured for the synthesized dental nanocomposite resins. Different superscript indicates statistically significant difference (*p* < 0.05).

Dental Nanocomposite Resin	Flexural Modulus (GPa) Mean (SD)	Flexural Strength (MPa) Median (IQR)
S.MPS 60	2.69 (0.43) ^a,c,d^	39.75 (34.59, 56.09) ^a^
S.MPS/Nanomer 55/5	1.71 (0.41) ^b,d^	32.66 (32.17, 35.39) ^a,b^
S.MPS/Nanomer 50/10	2.61 (0.46) ^c,d^	41.04 (32.85, 46.37) ^a^
S.MPS/Nanomer 40/20	2.50 (0.40) ^d^	37.88 (33.91, 39.39) ^a^
S.MPS/Nanomer 30/30	2.03 (0.38) ^b,c,d^	32.57 (30.86, 36.10) ^a,b^
S.MPS/CTAC 50/10	1.85 (0.46) ^b^	33.22 (22.80, 37.97) ^a,b^
S.MPS/S.CTAC 50/10	2.10 (0.50) ^a,b,c,d^	22.46 (15.12, 28.85) ^b^

## Data Availability

The data presented in this study are available from the corresponding author upon request.
